# Telemedicine Guidelines in South East Asia—A Scoping Review

**DOI:** 10.3389/fneur.2020.581649

**Published:** 2021-01-13

**Authors:** Mohamad Intan Sabrina, Irma Ruslina Defi

**Affiliations:** ^1^Neurorehabilitation Unit, Department of Rehabilitation Medicine, Hospital Rehabilitasi Cheras, Kuala Lumpur, Malaysia; ^2^Tung Shin Hospital, Kuala Lumpur, Malaysia; ^3^Faculty of Medicine, Hasan Sadikin General Hospital, Universitas Padjadjaran, Bandung, Indonesia

**Keywords:** universal, low to high income countries, guidelines, telemedicine, South East Asia

## Abstract

**Background:** Telemedicine is a useful tool to deliver healthcare to communities in low- to high-income countries, especially in the coronavirus disease 2019 pandemic era. Guidelines on telemedicine would assist healthcare providers in delivering healthcare services based on local circumstances.

**Objective:** To explore and compare guidelines on telehealth and telemedicine in South East Asian countries.

**Methods:** Electronic databases such as Google, PubMed, and Cochrane reviews were searched for articles using keywords such as “telemedicine” OR “telehealth” OR “eHealth” OR “telemedis” AND “guidelines” AND “South East Asia” OR “Malaysia” OR “Singapore” OR “Indonesia” OR “Thailand” OR “Vietnam” published up to 2020. Inclusion criteria were full articles and gray materials (i.e., policy statements, advisories, blueprints, executive summaries, and circulars) related to telemedicine guidelines. No language restrictions were imposed. Only the first 100 Google searches were included for eligibility based on its relevance to telemedicine guidelines. Exclusion criteria were abstracts, duplicate publications, blogs, news articles, promotional brochures, conference proceedings, and telemedicine projects unrelated to telemedicine guidelines.

**Results:** A total of 62,300 articles were identified through the search engines (Google 62,203, PubMed 77, and Cochrane 20) and six articles from additional sources. Sixty-eight full-text articles fulfilled the inclusion criteria, but only 24 articles contained some form of guidelines on telemedicine: Indonesia (nine), Malaysia (seven), Singapore (five), Thailand (two), and Vietnam (one). There were six laws, six advisory guidelines, five policy statements, and two circulars (regulations) issued by either the Ministry of Communication and Multimedia, Ministry of Health, or Medical Councils from the respective countries. Issues addressed were clinical governance (100%); information and communication technology infrastructure (83.3%); privacy, storage, and record-keeping (77.8%, respectively); ethics and legal (77.8%); security and safety (72.2%); definitions and applications of telemedicine (72.2%); confidentiality (66.7%); licensing (66.7%); identification (55.6%); cost of information and communication technology infrastructure (55.6%); reimbursement (16.7%); mobile applications (11.1%); and feedback and choices (5.6%). The Singapore National Telemedicine Guidelines contained the most domains compared with other guidelines from South East Asia.

**Conclusions:** Although there can be no “one-size-fits-all” telemedicine guideline, there should be a comprehensive and universal telemedicine guideline for any country to adapt based on the local context. Details on patient-identification, data ownership, back-up, and disposal; transregional cybersecurity laws and ways to overcome the limitations of telemedicine compared with face-to-face consultations should be outlined clearly to ensure uniformity of telemedicine service and patient safety.

## Introduction

Telemedicine, telehealth, or eHealth is the delivery of health-care services using information and communication technology (ICT) in the diagnosis, treatment, and prevention of disease or injuries, research, evaluation, and education for health-care providers and their communities (1).

Telemedicine is an efficient and cost-effective way to deliver acute, chronic, primary, and specialty care (2–5). However, the overall uptake of telemedicine has been slow among health-care providers globally, as it has been an optional rather than mainstream form of health-care delivery before the coronavirus disease 2019 (COVID-19) pandemic (6). Common barriers include technically challenged staff, cost, lack of high-speed internet (7, 8), conflicting health system priorities (8), and lack of political will (9).

Recent policy changes during the COVID-19 pandemic (10, 11) have reduced barriers to telemedicine. Advances in digital technology have expanded mobile health (mHealth) (8) applicability from providing health care in remote communities (12–14) to situations where face-to-face consultation is neither safe (6) nor practical (8). The use of mHealth in South East Asia (SEA) has increased exponentially in the last decade as it is the world's fastest-growing market for digital economy (8). The COVID-19 pandemic has spurred the growth of telemedicine from telephone consultations to a spectrum of ICT applications. The diversity in telemedicine practice across countries calls for uniformity in guidelines and standards (15). Payers, regulators, and policymakers would refer to guidelines and legislations, especially if telemedicine were integrated into existing policies and standard care. This scoping review aims to compare telemedicine guidelines in SEA, as the region shares common social and economic conditions.

## Objective

This scoping review aims to explore and compare guidelines on telehealth and telemedicine in SEA countries.

## Methods

**Figure 1:**
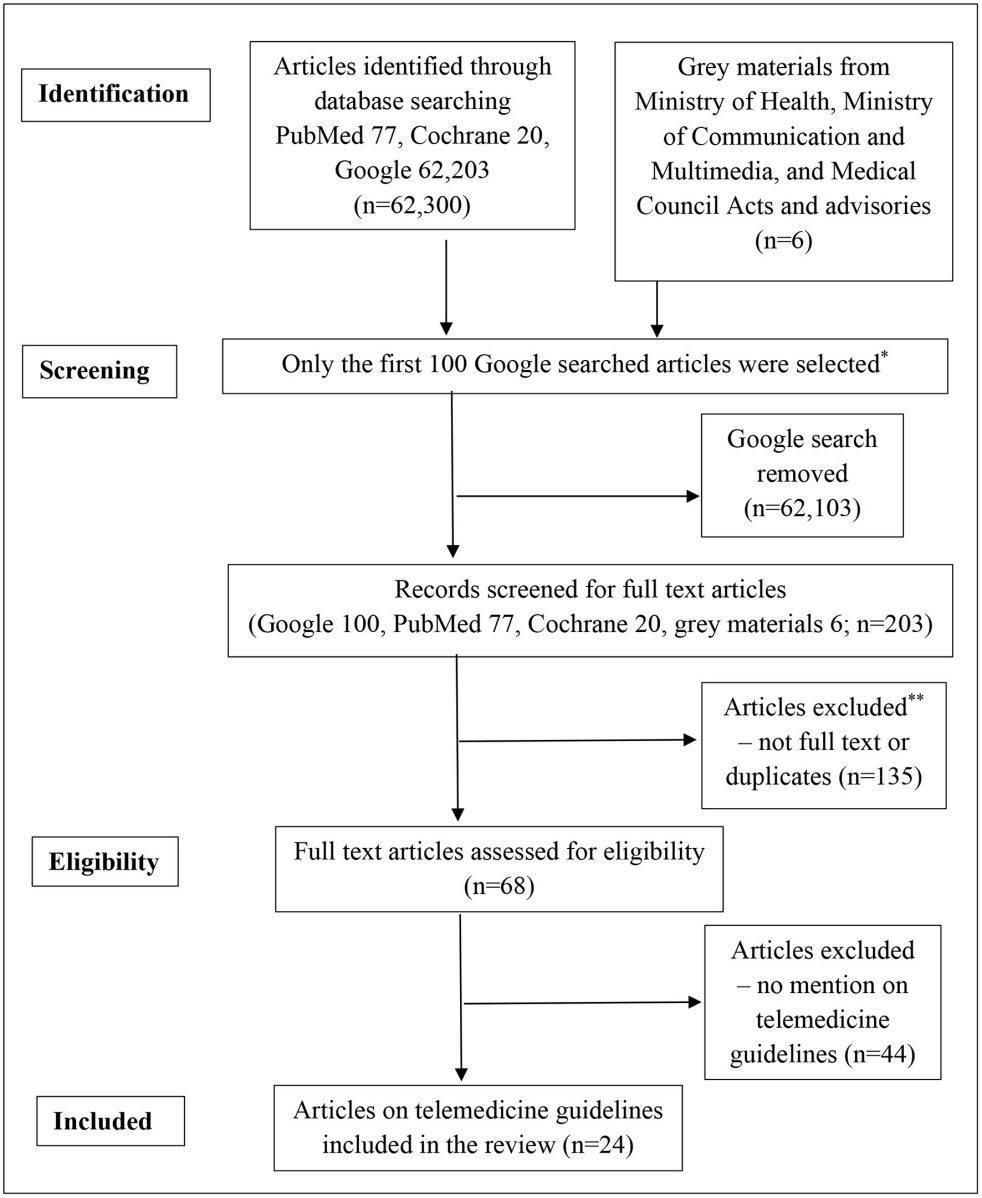
Flowchart of article selection. *Current conventional research methodology only includes the first 100 Google findings. **Blogs, news articles, promotional brochures, conference proceedings, and telemedicine projects unrelated to telemedicine guidelines.

Literature searches were conducted from 1 January to 7 May 2020 using PubMed, Cochrane, Embase, and Google search engines published up to 2020 (Figure 1). A combination of relevant MeSH and Emtree terms and keywords related to “telemedicine” OR “telehealth” OR “eHealth” OR “telemedis” AND “guidelines” AND “South East Asia” OR “Malaysia” OR “Singapore” OR “Indonesia” OR “Thailand” OR “Vietnam” was used. Records identified through database searching were screened for duplicates using the reference management software Mendeley. Additional sources were obtained through advisories and guidelines issued by the Ministry of Health and Medical Councils of Malaysia, Indonesia, Singapore, Vietnam, and Thailand during the COVID-19 pandemic from March 2020 to May 2020. Medical device and multimedia acts were identified through the Google search engine.

Inclusion criteria were full-text articles and gray materials (i.e., policy statements, advisories, blueprints, executive summaries, and circulars) related to telemedicine guidelines. No distinction was made to differentiate between advisories, guidelines, regulations, or laws. Only the first 100 Google searches were included for eligibility, as searches beyond 100 articles were repetitive and were not related to telemedicine guidelines. No language restrictions were imposed. Translations of original articles into English were included if they fulfilled the eligibility criteria. Only full-text articles were included, and duplicates were removed.

Exclusion criteria were abstracts, promotional brochures, blogs, news articles, conference proceedings, and specific telemedicine projects unrelated to telemedicine guidelines. Eligible studies were assessed for domains similar to a study published by Mars et al. (16) on WhatsApp guidelines. The domains were categorized into three main themes:

- Clinical aspects of telemedicine—definitions, clinical governance, applications, and international service.
- Ethical and legal issues—confidentiality, privacy, security, consent, identification, authentication, licensing, and cyber laws.
- Technical and operational issues—record keeping, data storage, phone stewardship, cost, billing or reimbursement, mHealth Apps, feedback, and choices offered to telemedicine users.

Articles retrieved from the literature search were screened at the title level by the first author ISM. Two reviewers (IRD and NHB) independently verified the accuracy and eligibility of the full-text articles. Any disagreement in the selection process was resolved by a consensus and consulting a third reviewer (PSR).

## Results

A total of 62,300 articles were identified through the search engines (Google 62,203, PubMed 77, and Cochrane 20) (see Figure 1). Six telemedicine and information technology laws were obtained from the Ministry of Health and Ministry of Communication and Multimedia of Malaysia and Indonesia. Searches from the Cochrane Library found no systematic reviews on telemedicine guidelines. Sixty-eight full-text articles fulfilled the inclusion criteria, but only 24 articles published from 1997 to 2020 contained some form of guidelines on telemedicine in SEA: Indonesia (nine), Malaysia (seven), Singapore (five), Thailand (two), and Vietnam (one).

Most articles were advisories (10, 17), policy statements (18–20), laws (21–26), circular (27), and blueprints (28–30) issued by the Ministry of Health (11, 20–24, 27–32) (50%) and Medical Councils (10, 18, 19) (16.7%). Five (20.8%) review articles (14, 33–36) discussed the current and future trends and best practices in telemedicine. Only Singapore (21), Malaysia (22–25), and Indonesia (26) have laws on telemedicine and the dissemination of electronic information. Table 1 shows a list of articles on telemedicine guidelines in SEA countries. The domains contained in the telemedicine guidelines are summarized in Table 2.

**Table 1:** Summary of telemedicine guidelines in South East Asian countries.

**No**.	**Article Title**	**Author(s)**	**Country of origin, year**	**Scope/domain/element**	**Type of article**	**Main findings**
1.	National Telemedicine Guidelines for Singapore	National Telemedicine Advisory Committee	Singapore, 2015	Clinical, ethical, technical, and operational aspects of telemedicine and practitioners.	Advisory guidelines	Definitions of telemedicine, consent, identification confidentiality, privacy, security, record keeping, data storage, licensing, clinical governance, ethics, legal issues, cost, user feedback, and options.
2.	Ethical code and ethical guidelines 2016 Edition	Singapore Medical Council	Singapore, 2016	Clinical, ethical, technical, and operational aspects of telemedicine and health practitioners.	Advisory guidelines	Guideline on “remote consultation” (telemedicine) for initial and continuing care consultations, confidentiality, identification, record-keeping, international service, licensing, fees, and clinical governance.
3.	Ethical code and ethical guidelines 2002	Singapore Medical Council	Singapore, 2002	Clinical, ethical, technical, and operational aspects of telemedicine and health practitioners.	Advisory guidelines	Guideline on remote initial consultations and remote consultations (telemedicine) in continuing care, confidentiality, identification, record-keeping, licensing, and clinical governance.
4.	Health care Services Act (HCSA)	Ministry of Health, Singapore	Singapore, 2021–2022	Clinical, ethical, and legal aspects of telemedicine licensing, technical, and operational standards.	Act	Licensing, ethics, quality assurance, and clinical governance of health-care service providers (Principal Officer and Clinical Governance Officer).
5.	Regulatory Guideline for Telehealth Products	Medical Devices Branch, Health Sciences Authority	Singapore, 2019	Clinical, ethical, legal, technical, and operational standards of telehealth products.	Policy statement/guidelines	Definition of telehealth, telehealth product licensing, clinical governance, and infrastructure.
6.	Layanan Telemedis di Indonesia: Keniscayaan, Risiko, dan Batasan Etika	Prawiroharjo P., Pratana P., and Librianty N.	Jakarta, Indonesia, 2019	Clinical, ethical, legal, technical, and operational aspects of telemedicine and health practitioners.	Review article	Clinical governance, ethics, confidentiality, security, privacy, legal, cost, data storage, record keeping, and infrastructure.
7.	Kebijakan Pengembangan Tele-Medisin Di Indonesia	Bernhard HS	Jakarta, Indonesia, 2015	Clinical, ethical, legal, technical, and operational aspects of telemedicine and health practitioners.	Review article	Confidentiality, record keeping, data storage, clinical governance, ethics, legal issues, human resource, infrastructure, international service, spectrum of telemedicine services, and cost.
8.	Peraturan Konsil Kedokteran Indonesia Nomor 74 Tahun 2020 Tentang Kewenangan Klinis dan Praktik Kedokteran Melalui Telemedicine Pada Masa Pandemi Corona Virus Disease 2019 (Covid-19) di Indonesia	Supriyanto B. and Ekatjahjana W. Indonesian Medical Council	Indonesia, 2020	Clinical, ethical, technical, and operational aspects of tele-consultation and tele-practice.	Advisory guidelines	Definitions, consent, clinical governance, ethics, confidentiality, record keeping, data storage in medical and health facilities (Fasyankes), imbursement, and prohibitions.
9.	Permenkes No. 20 Tahun 2019 tentang Penyelenggaraan *Telemedicine* antar Fasilitas Pelayanan Kesehatan	Moeloek NF and Ekatjahjana W. Ministry of Health, Republic of Indonesia	Indonesia, 2019	Clinical, technical, and operational aspects of telemedicine services.	Advisory guidelines	List of definitions related to telemedicine, diagnostics, record keeping, infrastructure and Apps licensing, human resource, leadership, ethics, identification, consent, confidentiality, clinical governance, task of health-care facilities that provide telemedicine, cost, reimbursement, and funding regulation in Indonesia.
10.	Peraturan Badan Pengawas Obat dan Makanan Nomor 8 Tahun 2020 Tentang Pengawasan Obat dan Makanan Yang Diedarkan Secara Daring	Ministry of Health, Indonesia	Indonesia, 2020	Clinical, ethical, legal, technical, and operational aspects of tele-pharmacy.	Policy statement/guidelines	Definition, privacy, confidentiality, consent, identification, clinical governance, ICT infrastructure, ethics, legal, licensing, and mHealth.
11.	Kajian Tekno-Ekonomi pada *Telehealth* di Indonesia (*Techno-Economic Study on Telehealth in Indonesia*)	Sri A and Kautsarina	Jakarta Pusat, Indonesia, 2017	Clinical, technical, and operational aspects of telemedicine.	Review article	Capital and operational expenditures of telehealth programs, definition, infrastructure, clinical governance, record keeping, and data storage.
12.	Overview of Telemedicine Activities in Indonesia: progress and constraints	Andriyan B. S., Sastro-kusumo U., Tati L.R. et al.	Bandung, Indonesia, 2004	Clinical, technical, and operational aspects of telemedicine.	Review article	History, present, and development of telemedicine in Indonesia, international service, and parties involved in telemedicine.
13.	Surat Edaran Nomor HK.02.01/Menkes/303/2020 Tentang Penyelenggaraan Pelayanan Kesehatan Melalui Pemanfaatan Teknologi Informasi Dan Komunikasi Dalam Rangka Pengahan Penyebaran Corona Virus Disease 2019 (Covid-19)	Ministry of Health, Indonesia	Indonesia, 2020	Clinical, ethical, technical, and operational aspects of telemedicine and health practitioners.	Circular/guidelines	Definitions, clinical governance, privacy, security, record keeping, infrastructure, ethics, and cost.
14.	Law of the Republic of Indonesia Number 11 of 2008 Concerning Electronic Information and Transactions Undang-undang Republik Indonesia Nomor 11 Tahun 2008 Tentang Informasi Dan Transaksi Elektronik.	Ministry of Law and Human Rights, Indonesia	Indonesia, 2008	Legal, technical and operational aspects of information and electronic transactions.	Act	Definitions, governance, security, record keeping, storage, distribution, infrastructure, legal
15.	Malaysian Medical Council Advisory on Virtual Consultation (during the Covid-19 pandemic)	Malaysian Medical Council	Malaysia, 2020	Clinical, ethical, legal, technical, and operational aspects of telemedicine and health practitioners.	Advisory guidelines	Definition of “virtual consultation” (telemedicine), ethics, patient and health-care practitioner identification, consent, ethics, clinical governance, and legal.
16.	Telemedicine Flagship Application: Malaysia's Telemedicine Blueprint Leading Healthcare into the Information Age	Ministry of Health, Malaysia	Malaysia, 1997	Clinical, legal, technical, and operational aspects of telemedicine.	Policy statement	List of definitions and applications of telemedicine, infrastructure, data storage, record-keeping, legal, and cost.
17.	HIMS Blueprint – toward excellence in Health Information Management	Health Informatics Center, Planning Division, Ministry of Health Malaysia	Malaysia, 2013	Technical and operational aspects of telemedicine (Health Information Management and Support Services) in MoH and related agencies.	Policy statement	Confidentiality and privacy, security and data protection, consent, user access, role of stakeholders, infrastructure support, health informatics standards, capacity, and capability building.
18.	Telemedicine Act 564	Ministry of Health, Malaysia	Malaysia, 1997	Clinical, ethical, legal, technical, and operational aspects of telemedicine and health practitioners.	Act	Definitions of telemedicine and health-care practitioners, confidentiality, identification, record keeping, data storage, international service, ethics, and legal issues.
19.	Medical Device Act (737) 2012	Medical Device Authority, Malaysia	Malaysia, 2012	Clinical, legal, technical, and operational aspects of medical devices.	Act	Definition and classification of medical device, therapeutic and diagnostic digital applications and data, clinical governance, licensing, maintenance, legal issues, and fees.
20.	Medical Device Authority Act (738) 2012	Medical Device Authority, Malaysia	Malaysia, 2012	Clinical, legal, technical, and operational aspects of control and regulation of all matters relating to the medical device, the industry, and its activities.	Act	Clinical governance, licensing, infrastructure, research, and training, legal and funding issues.
21.	Laws of Malaysia Act 588 Communications and Multimedia Act 1998	Ministry of Multimedia and Communication, Malaysia	Malaysia, 1998	Legal and technical aspects of information sharing on the internet.	Act	Governance, infrastructure, licensing, legal, and intellectual property.
22.	eHealth Strategy, Ministry of Public Health (2017–2026)	Ministry of Public Health, Thailand	Thailand, 2017	Clinical, technical, and operational aspects of telemedicine and health innovation.	Policy statement	Definitions and benefits of eHealth; electronic health record and eHealth uptake; ICT and telemedicine infrastructure and readiness; and 5-year eHealth action plan compliant with the digital economy.
23.	Ethics, social medical and e-health in Thailand	Suttisak J.	Thailand, 2015	Clinical, ethical, legal, technical, and operational aspects of telemedicine and social media.	Literature review	Definitions, social media Apps, privacy, security, confidentiality, identification, legal, ethics, record keeping, storage, clinical governance, infrastructure.
24.	Regulating the management of Distance Medicine (Circular No. 49/2017/TT-BYT dated December 28, 2017 on telemedicine)	Ministry of Health, Vietnam	Vietnam, 2017	Clinical, ethical, legal, technical and operational aspects of distance medicine within Vietnam and Vietnamese medical facilities from abroad	Circular/ guidelines	Definition of “distant medicine” (telemedicine), confidentiality, ethics, data storage, clinical governance, licensing, infrastructure, cost, and international service. Distance medicine activities may only occur at licensed facilities; storage and compression of images must be on a 4 Mbps or faster line.

**Table 2:** Domains contained in the telemedicine guidelines of South East Asian countries.

**Domains**	**Singapore (*n* = 5)**	**Malaysia (*n* = 7)**	**Indonesia (*n* = 9)**	**Thailand (*n* = 2)**	**Vietnam (*n* = 1)**
**Clinical**					
Definitions of telemedicine	Yes	Yes	Yes	Yes	Yes
Clinical governance	Yes	Yes	Yes	Yes	Yes
Restrictions	Yes	Yes	Yes	No	No
International service	Yes	Yes	Yes	No	Yes
**Ethical and legal**					
Medical ethics	Yes	Yes	Yes	Yes	Yes
Legislation	Yes	Yes	No	No	No
Consent from users	Yes	Yes	Yes	No	No
Confidentiality and privacy	Yes	Yes	Yes	Yes	Yes
Identification/authentication (providers, patients)	Yes	Yes	Yes	No	No
**Operational and technical**					
Data security and stewardship	Yes	Yes	Yes	Yes	Yes
Record keeping and data storage	Yes	Yes	Yes	Yes	Yes
Licensing of health-care practitioners	Yes	Yes	Yes	No	Yes
Licensing of health-care facilities	Yes	Yes	Yes	No	Yes
Licensing of telehealth products (mHealth, Apps)	Yes	Yes	Yes	Yes	No
Licensing of traditional and complementary medicine	No	No	Yes	No	No
ICT infrastructure	Yes	Yes	Yes	Yes	Yes
Internet speed requirement	No	No	Yes	No	Yes
Human resource	Yes	Yes	Yes	Yes	Yes
Cost of ICT infrastructure, training, human resource	Yes	Yes	Yes	Yes	Yes
Reimbursement/service fee	Yes	No	Yes	No	Yes
Feedback from users	Yes	No	No	No	No
Choices offered to users	Yes	No	No	No	No

*ICT, Information and Communication Technology; EMR, electronic medical records; HIMS, Health Information Management System; mHealth, mobile health, Apps, phone applications*.

### Clinical Aspects of Telemedicine

#### Definitions of Telemedicine and Telehealth Products

Several terminologies were used to define telemedicine, such as “remote consultation,” (18, 19) “virtual consultation,” (10) “distant medicine,” (27) “e-Health and digital technologies” (30), and “cybermedicine and telemedis” (33). Although most guidelines include medical activities and services involving ICT as part of telemedicine (10, 14, 17–24, 28, 29, 31–36), the latter refers to long-distance medical service in Thailand (30) and Vietnam (27).

Telehealth products may be defined as any instrument, appliance, software, or similar applications intended by the manufacturer to be used alone or in combination for the purpose of diagnosis, prevention, monitoring, treatment, or support of the anatomy or physiological process (28, 29, 32). Devices that monitor biometrics or lifestyle habits are not considered telehealth products in Singapore (32). Other SEA countries do not have clear definitions of telehealth products or the scope of its use in telemedicine services within or beyond their country.

#### Applications and Restrictions in Telemedicine

Almost all guidelines from SEA countries outlined the spectrum of telemedicine from real-time telehealth such as tele-consultation (10, 14, 17–20, 27, 33–36), tele-treatment (17, 34), tele-surgery (34, 35), tele-rehabilitation (34), tele-pharmacy (17, 31, 34), tele-radiology (17, 27), tele-pathology (17, 27, 34), tele-diagnostic (14, 27) to remote patient monitoring (17, 27, 34), tele-support (17), tele-coaching (34), tele-nursing (34), tele-homecare (17, 34), tele-rehabilitation (34), tele-collaboration (14, 17, 35), and tele-education (14, 17, 27, 28, 33, 34). The scope of telemedicine services depends on the existing needs and policies of the organization and medical councils. Most guidelines issued by the medical councils in Malaysia (10), Indonesia (11, 20), Singapore (17–19), and Vietnam (27) tend to regulate health-care professionals rather than the technologies, platforms, or type of telemedicine services (10). Telemedicine can only be conducted at registered health facilities in Indonesia (11, 31) and Vietnam (27). It is unclear how such rules apply to mobile applications in the respective countries. The guidelines from Singapore, Malaysia, and Thailand are less restrictive on where and how telemedicine activities can be carried out.

Tele-consultation is only permitted for patients already known to the health-care practitioners and/or as part of a continuation of care in Singapore (17–19) and Malaysia (10). New referrals, emergency cases, and invasive procedures require face-to-face consultation and physical presence in Malaysia (10) and Indonesia (11, 20). Telemedicine guidelines from Thailand and Vietnam made no mention of patient-selection, technologies, or platforms for tele-consultation.

Tele-pharmacy (e-pharmacy) is a mode of pharmacy service that utilizes technology to improve access, such as online prescription and/or counseling and dispensary *via* postal services (32). Prescriptions are transmitted electronically through a closed-loop electronic interface from the licensed practitioner to the licensed pharmacist on secured online platforms (31, 32, 36). The medicines are then delivered directly to the patients *via* postage or collected at designated pharmacies. Only Singapore and Indonesia have guidelines on electronic prescriptions, which can only be performed by licensed health-care medical practitioners and must not include narcotics and psychotropic drugs (31, 32). The telemedicine guideline from Vietnam permits a spectrum of telemedicine services, from diagnosing to prescribing appropriate treatment (27). However, it is unclear if the latter included tele-pharmacy. At present, online prescriptions other than narcotics and psychotropic drugs are only permitted as a continuation of care in Malaysia (10).

#### Clinical Governance and International Service

Most telemedicine guidelines have elements of clinical governance (10, 11, 17, 27, 30). Only registered health-care practitioners are allowed to practice telemedicine in their respective countries (10, 11, 17, 27, 33–36). International telemedicine service should be delivered in collaboration with the health-care provider licensed in the patient's country (10, 17, 22). The telemedicine guidelines from Singapore, Indonesia, and Vietnam have provisions for patients to receive treatment from abroad, including overseas medical facilities, agencies, organizations, and individuals related to telemedicine activities (17, 27, 31). The latter implies a spectrum of services, from referring a patient to diagnosing, imaging, operating, and prescribing appropriate treatment (27).

### Ethical and Legal Aspects of Telemedicine

#### Ethics

Health-care practitioners must adhere to the same ethical standards and code of conduct, whether the telemedicine service is sourced locally or from abroad (10, 17, 22, 27). Distance medical advice may only be given within the scope of the specialty outlined in the practicing certificate of the provider for the advice (10, 17, 27, 36). The medical council jurisdiction of each country only applies within its country (10, 19, 20). Physicians must ensure that proper liability protection is in place to provide indemnity for malpractice (10, 19).

#### Legislation and Licensing of Telehealth Products

Singapore and Malaysia are the only SEA countries with separate laws to regulate telemedicine practices (21, 22) and telehealth products (23, 24, 32). The Telemedicine Act from Malaysia (22) focuses on regulating health-care professionals practicing telemedicine. The Healthcare Services Act of Singapore (21) is designed to ensure patient safety through proper licensing of medical institutions and professionals providing telemedicine services. Laws that apply to all telehealth products (23, 24, 32) are separate from the practice of telehealth services (21, 22) in Singapore and Malaysia. Devices that are intended to promote wellness do not fall under the purview of telehealth laws in Singapore (32). Other SEA countries (26, 27, 34, 36) do not seem to have a clear distinction between the telehealth products and/or its use in telemedicine.

Cyber laws such as the Digital Signature/Contract Act, Computer Crime Act, Multimedia Intellectual Property Act, and Electronic Government Act of Malaysia (25) and Indonesia (26) are meant to regulate ICT copyrights and to prevent dissemination of false information on the internet. Such laws may be applied in telemedicine but need to be reviewed for its relevance (28). To date, there is no transregional telemedicine treaty in the SEA region.

None of the telemedicine guidelines or cyber laws in SEA countries outlined the procedures or best practices on tele-consultations using ICT devices such as telephone consultation or smartphone applications (Apps). Tele-consultations can only be conducted by institutions registered with the Ministry of Health in Indonesia (20) and Vietnam (27). The Singapore National Telemedicine Guidelines (NTG) deemed teleconsultations *via* mHealth and Chatbots as inappropriate (17). Malaysia and Singapore are the only countries with guidelines on differences between medical advice from advice for wellness (10, 32).

#### Informed Consent and Options

Other than Thailand, most telemedicine guidelines contain some elements of informed consent before the commencement of telemedicine (10, 11, 17–20, 22, 27, 31). The manner of obtaining informed consent must adhere to the medical ethics of the respective medical councils (10, 11, 17, 27). Implied consent is consent that is not expressly granted by a person but perceived by the service provider that the person has agreed to the service. Explicit consent may be obtained in verbal or written formats (10). Patients should be informed of the possible intended purpose(s) on how the data will be used and the available options before proceeding with telemedicine (17). The guidelines from Malaysia and Vietnam mentioned that the provision of telemedicine is voluntary (10, 27). Patients can withdraw from receiving telemedicine at any stage (10, 17). Telemedicine limitations must be explained to the patient (10, 11, 17), as clinical assessments may be limited to audio and visual information (10, 17). In-person assessments should be arranged if telemedicine medium were inadequate (10, 11, 17).

#### Privacy, Confidentiality, and Data Security

Most telemedicine guidelines have policies to protect the privacy of patient information (10, 11, 17–20, 22, 27–29). However, details on data handling and stewardship, information-sharing, and record-keeping vary from one country to another. Indonesia and Vietnam only permit telemedicine to be conducted *via* internet systems at registered health facilities (11, 27) to ensure data security and confidentiality. Indonesia, Malaysia, and Thailand have policies on data management and data security using government information networks (14, 28, 30). Most guidelines state that the responsibility of data security falls on individual telemedicine providers (17, 27).

#### Identification and Authentication

Telemedicine providers must ensure that the identities of the parties involved, place of practice, and registration status are made known to the patient and confirm the identity of the patient at each consultation (10, 17). None of the telemedicine guidelines in SEA outlined the patient-identification and authentication processes in detail.

### Operational and Technical Aspects of Telemedicine

#### Record-Keeping and Data Storage

Telemedicine consultations and activities must be recorded, either as manual transcripts (10, 11) or electronic medical record (10, 17, 27, 28, 30, 36) by the telemedicine provider. Records should be kept at their respective facilities (11, 27) for easy retrieval (10, 17) and audit trails (17). Medical images and video footage should be stored in the database of the telemedicine provider (11, 27). The Vietnamese guideline recommends a minimum storage capacity of 10 years (27). Other SEA guidelines did not specify any minimum requirements.

#### Data Ownership and Management

Malaysia and Thailand have policies on data management *via* their respective Health Information Management Systems (28, 30). The responsibility of data stewardship falls on the respective telemedicine providers (11, 17, 27, 28, 30). None of the telemedicine guidelines specifically addressed the issues of data ownership, back-up, disposal, deletion, or viral attacks. Unless telemedicine is conducted *via* registered and licensed health-care facilities with secure networks (11, 27, 31), it is unclear how data ownership, privacy, and security can be regulated. Current telemedicine guidelines and cyber laws in SEA countries have not addressed cybersecurity breaches and attacks (25, 26) in detail.

#### Information and Communications Technology Infrastructure

ICT is a major component in telemedicine (10, 11, 14, 17, 27, 30) and may be classified into technology and equipment (14, 17, 28, 29, 32–35). ICT infrastructure must satisfy confidentiality, safety, data security (17, 27, 28), and interoperability standards for effective and efficient delivery of telemedicine services and user satisfaction (17). The NTG from Singapore is the only guideline to mention scalability, maintenance of technology, equipment calibration, end-of-life, and e-waste disposal (17). ICT equipment and technology may require upgrades and replacement to suit evolving technology and needs (17, 35).

Vietnam is the only country to state the minimum broadband speed for teleradiology consultation and tele-education, which are 4 and 2 Mbps, respectively (27). Other SEA countries did not specify any high-definition technology. Wireless local area network, satellite technologies, and telecommunication networks are used in Indonesia, Malaysia, Thailand, and Vietnam because of its affordability and easy access in rural communities (14, 28, 34). ICT hardware and connectivity may not be available in all health-care facilities (11, 14, 33–36) due to the high cost of setting up telemedicine infrastructure (34, 37). Health-care professionals and patients use a telephone, short messaging system, multimedia messaging system, iMessage, WhatsApp, Chatbots, email (17, 34), and other audiovisual platforms with varying broadband speed (34).

#### Human Resources

Almost all of the telemedicine guidelines from SEA have policies on human resources to deliver telemedicine (10, 17, 21, 27, 28). Such policies should be reviewed regularly due to the evolving nature of the field (10, 11, 31). Health-care providers should possess adequate training and competency to manage patients through telemedicine (10, 17) and act within the capacity of their qualifications and medical registration (10, 11, 17–20, 27). Health facilities should provide training and technology transfer in telemedicine, which can be developed through structured on-the-job training (17) and tele-education (27, 33, 34). Organizations offering telemedicine services should have strategies to retain personnel, including reviewing compensation to ensure that it is fair and equitable (17).

#### Costs of Telemedicine

The costs of telemedicine depend on the country's gross domestic product and spending on eHealth (14, 28, 30, 35). Factors to consider comprise the types of telehealth programs, number of health facilities providing telemedicine services, ICT infrastructure, capital expenditure, and operational expenditure (35). Telemedicine expenditures should be incorporated into current and planned funding structures (28, 35). Opex cost is projected to peak in the fourth year of a 5-year cycle due to internet subscription, maintenance and replacement of technology and equipment, and training health-care professionals in Indonesia (35).

Other cost issues include cost–benefits of health promotion, disease prevention, and early intervention; availability and utilization of health-care services in the community; effect of telemedicine on the cost, type, size, and distribution of health-care facilities (28); and resource allocation to achieve widespread implementation of telemedicine, financing insurance products, and reimbursement linked to telemedicine (17). The costs of providing telemedicine in health facilities in Malaysia (10, 28, 29), Singapore (17), Indonesia (20, 34, 35), Thailand (30), and Vietnam (27) are borne by the respective health-care service providers.

#### Reimbursement and Fees

Telemedicine is provided for free in Malaysia and Thailand, as there is no billing structure at the time of writing (10, 22, 30). IT system operating costs and other extra costs serving provision of telemedicine shall be paid in accordance with regulations of the law in Vietnam (27), Indonesia (11), and Singapore (17). None of the telemedicine guidelines from SEA contained the billing structure or which health-care providers are allowed to be reimbursed for their services.

#### Feedback and Evaluation

The NTG from Singapore (17) is the only guideline to mention quality improvement activities, impact on cost and accessibility of care, patient outcome and satisfaction, provider satisfaction, technical quality of service, and quality of communication. Telemedicine guidelines from Malaysia (10, 22) and Indonesia (20, 33) emphasized good patient–doctor communication to avoid medicolegal consequences.

## Discussion

In general, most SEA countries have guidelines on telemedicine with varying degrees of breadth and depth. Most of the SEA guidelines focused on ethical and clinical aspects of telemedicine, with less emphasis on the technology or platform to deliver the service. The NTG from Singapore (17) is the most comprehensive guideline in the SEA region and comparable with other telemedicine guidelines around the world (16, 38–44). Much is needed to standardize telemedicine guidelines so that it could be applied to the local context (1). Areas that need standardization are terminologies, restrictions, applications, legislation, and billing of telemedicine. Regulations on traditional and complementary medicine (TCM) should also be included in telemedicine guidelines, given that TCM plays an integral part in the national health-care system in Asian countries (37, 45–51). Indonesia is the only SEA country to have a policy on online TCM activities (31).

Telemedicine guidelines are meant to give practical advice to medical practitioners so that telemedicine is integrated into existing health systems (39, 42–44). Some guidelines are mandatory, whereas others are only advisories and not legally binding. The Telemedicine Act 1997 of Malaysia (22) serves to regulate and control the practice of telemedicine and matters connected therewith. Any person who practices telemedicine in contravention of the Act shall be liable to an RM 500,000 fine or imprisonment for a maximum of 5 years or both. None of the other SEA countries have specific laws on telemedicine, other than regulations for the registration of medical institution (21) and/or products intended for telemedicine services (31, 32). Most cyber laws in SEA countries were designed to protect intellectual property rights and to prevent the dissemination of false or classified information in their country (25, 26). However, these guidelines did not specify the boundaries of international mHealth services (16, 22–25). It is unclear if laws such as the Personal Data Protection Act and cyber-laws in the SEA countries (25, 26) had international jurisdiction for technology breach. The lack of uniformity in laws and regulations makes it difficult to enforce legislation in malpractice across borders (39, 52, 53). There should be international collaborations to deal with transregional jurisdiction over breach of security and other cyber-crimes.

Distance is no longer a prerequisite for telemedicine (6, 10, 11, 39) but a necessity to deliver health care (54–58). Key changes were made to existing telemedicine policies globally after the Covid-19 pandemic in 2020 (10, 11, 39, 57, 58). Telemedicine creates opportunities for international collaborations, data-sharing, and technology-transfer among health-care providers (1, 27, 41–43, 55, 56). ICT infrastructure for telemedicine has been expanded to social media (36, 39, 55, 56, 59) to cope with the dazzling speed of information-sharing and evolving technology. Issues such as privacy, confidentiality, data security, and ownership (16, 60) must be refined, particularly when it involves international data-sharing.

Network readiness index (NRI) (61) is a quantitative measurement used as a benchmark for telemedicine readiness. Countries with lower NRI scores lag in telemedicine due to limitations in ICT infrastructure and technical expertise. Population size, geographical landscape, and country income bracket may contribute to the disparities in telemedicine readiness. Singapore is a small high-income country with a population of 5.6 million (62) and ranks the second-highest NRI in 2019, after Sweden (61). Countries with lower NRI ranking, such as Indonesia and Vietnam, face more challenges to achieve high internet and smartphone penetration (63) for its 273.5 million and 97.3 million people (62), respectively. The lack of ICT infrastructure and challenging geographical landscape (14, 34) further complicate their network readiness and advancement in telemedicine.

Despite these shortcomings, most SEA countries invest in digital health solutions due to their potential in the digital economy (8, 28, 30, 34, 35, 59, 64, 65) and its usefulness during the COVID-19 pandemic (2, 6, 54–58). The lack of ICT infrastructure in most SEA countries has been overcome by using existing resources and free mHealth Apps offering affordable consultation fees (59, 65–67). Telemedicine is provided for free in public hospitals in Malaysia, Vietnam, and Thailand at the time of writing. A 15-min tele-consultation *via* mobile applications in Singapore costs between SGD12.50 (USD 8.96) to SGD25 (USD 17.92) compared with SGD13.20 (USD9.46) to SGD27 (USD19.35) for an in-person medical consultation at government-funded polyclinics (59, 66). In contrast, a 30-min teleconsultation in Indonesia may cost between 25,000 and 75,000 Rupiah (USD 1.5–USD 5.0) in Indonesia. Other than Singapore (67), most SEA countries do not have a national insurance scheme that includes telemedicine rebates or subsidies such as Medicare in Australia (43). Uniformity in telemedicine guidelines will facilitate insurers and policymakers to reimburse telemedicine services fairly within and across countries.

## Limitations of the Study

This review is limited to five countries in SEA and only focused on domains mentioned in most guidelines. Future studies should include other Asian countries such as India, China, and other Association of South East Asian Nations for a more representative overview (68). A more comprehensive review of insurance schemes and billing systems across countries will need to be undertaken to reimburse telemedicine services within and beyond the country (69).

A universal and generic guideline outlining the minimum standard for telemedicine should be set by the World Health Organization to be adapted and applied to the local context. Transregional telemedicine legislation would facilitate international cooperation in the scientific, legal, and ethical aspects of telemedicine.

This review also did not include the credentialing and training of human resources in detail, such as telemedicine services other than medical teleconsultation. Further studies should focus on specific telemedicine services such as tele-therapy or tele-diagnostics and outcome measures to improve the implementation of telemedicine. The COVID-19 pandemic forced us to relook at our health-care systems and adapt to changing consumer trends and requirements. By doing so, telemedicine may no longer be an option but be the standard of care.

## Conclusions

Although there can be no “one-size-fits-all” telemedicine guideline, there should be a comprehensive and universal telemedicine guideline for any country to adapt based on the local context. Details on patient-identification, data ownership, back-up, and disposal, transregional cybersecurity laws, and ways to overcome the limitations of telemedicine compared with face-to-face consultations should be outlined clearly to ensure uniformity of telemedicine service and patient safety.

## Author's Note

Telemedicine is an efficient and cost-effective tool to deliver acute, chronic, primary and specialty healthcare to communities. Recent policy changes during the Covid-19 pandemic have reduced barriers to telemedicine and expanded mobile health applicability, especially *in situations* where face-to-face consultation is neither safe nor practical. A universal telemedicine guideline would be useful for healthcare providers to refer to and adapt based on local context. The aim of this scoping review is to explore telemedicine guidelines in South East Asia and compare them to existing guidelines from other regions.

## Author Contributions

All authors listed have made a substantial, direct and intellectual contribution to the work, and approved it for publication.

## Conflict of Interest

The authors declare that the research was conducted in the absence of any commercial or financial relationships that could be construed as a potential conflict of interest.
